# Using the Chief Complaint Driven Medical History: Theoretical Background and Practical Steps for Student Clinicians

**DOI:** 10.15694/mep.2020.000017.1

**Published:** 2020-01-20

**Authors:** Richard J. Nierenberg

**Affiliations:** 1 Hackensack University Medical Center

**Keywords:** Learning theory, expertise, differential diagnosis, medical history, cognitive processes, chief complaint, medical students

## Abstract

This article was migrated. The article was marked as recommended.

A previous short report presented an approach to teaching a focused medical history in the emergency department by using a chief complaint directed differential diagnosis guided streamlined series of questioning. It was proposed that such an approach teaches clinical expertise. The current article presents a review of a robust literature in the acquisition of cognitive expertise, and specifically how novices become experts through the acquistion of increasingly relevant and pertinent information. The review traces the development of several concepts such as exemplars illness scripts, problem representations, the use of semantic qualifiers and shows how the current proposed method incorporates those approaches. The method is applied specifically to two patient chief complaints commonly encountered in the emergency deparment and suggests how this approach would be useful in developing diagnostic ability in student physicians.

## Introduction

**Figure 1.**
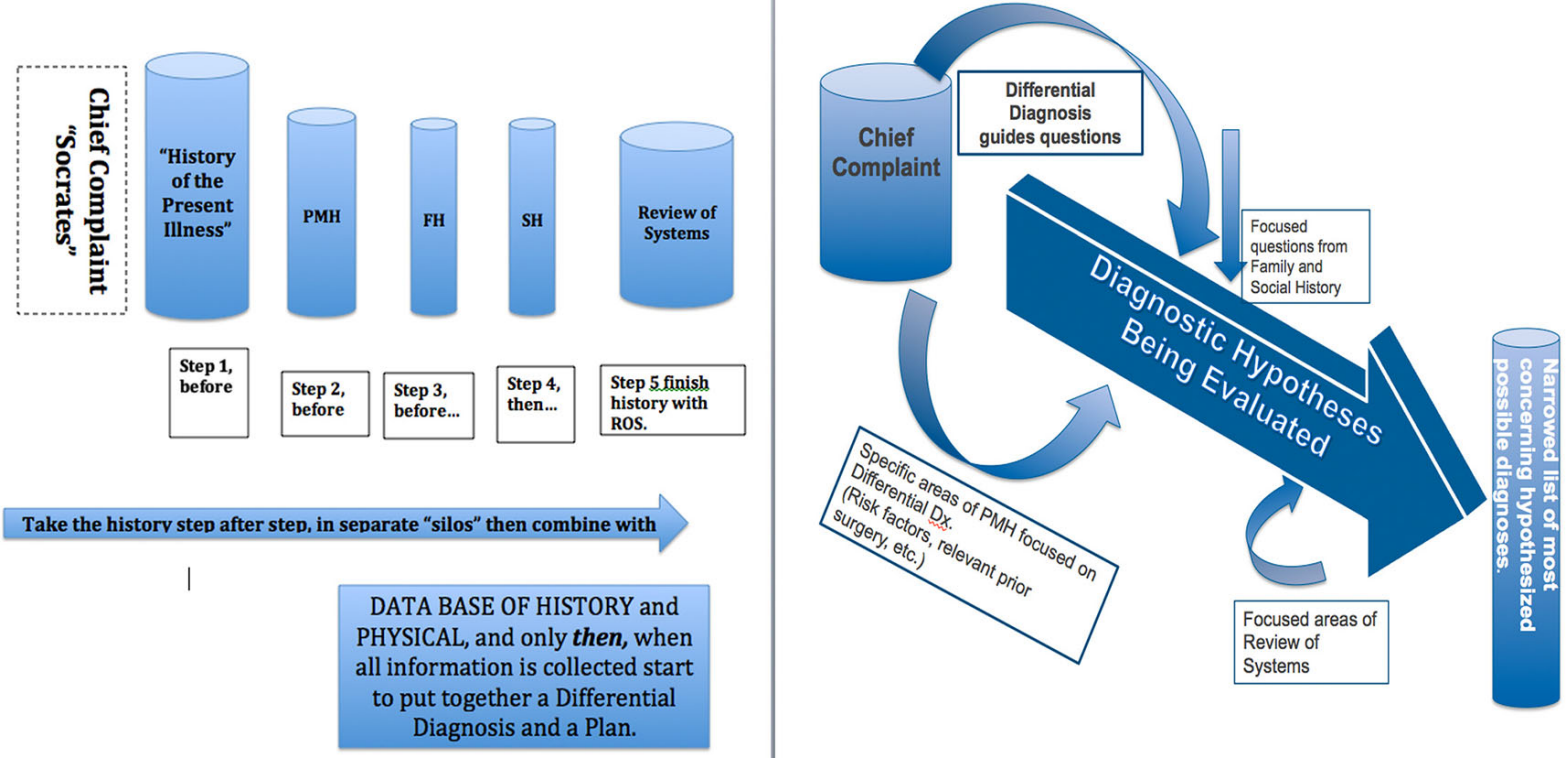
Comparison of The Traditional Medical History with Chief Complaint Driven Medical History

In 2017 the author published a short paper ( Nierenberg, 2017) proposing an approach to teaching medical students to obtain a concise, focused, pertinent and accurate history from patients in the Emergency Department. This approach differed from the traditional approach to obtain the history in sequential and ‘siloed’ categories of historical information. The history of the present illness and past medical history, for example, were suggested not to be gathered in the traditional sequential categories, but rather as part of a specific and directed problem solving process based on the development of a focused differential diagnosis rooted in the chief complaint. A conceptual diagrammatic presentation of this difference can be seen in Figure 1. This figure illustrates a conceptual change from gathering data in separate categories, to using the differential diagnosis to direct questioning of the patient.

Figure 1 contrasts the traditionally taught medical history on the left with the chief complaint driven history shown on the right. Traditionally the “history of the present illness” takes the chief complaint and details every component, often using the well known “Socrates” mnemonic, (site, occurrence, character and so on) before moving first to the comprehensive past medical history, the family history, sequential on to the review of systems and then combines to form a differential. The chief complaint driven history proposes that any chief complaint will generate a immediate list of several possible diagnostic entities. Each of those can be made more or less likely by a small set of specific questions drawn from elements of the traditional history of the present illness but also taken from past medical history, social history, and the like. The overriding guide is that every specific question asked is meant to take one closer to or further from the likelihood of a specific diagnostic entity under consideration. For example, with chest pain, the question “have you have had a heart attack” or “do you have hypertension” might well come before every aspect of the traditional HPI is satisfied, as might “do you have a history of blood clots or cancer” in consideration of the possibility of a pulmonary embolism.

This approach was proposed to work particularly well in the emergency department because in that setting there generally are a relatively limited number of specific complaints that bring patients to the provider. The need to efficiently obtain an accurate history, develop a clear differential diagnosis, and present this information and formulation to a supervising or collaborating physician is as important and pressing in the ED as in any medical situation ( Croskerry, 2002). Here the demand for a quick, focused and effective presentation has prompted some to propose that medical students be able to accurately present a case in as little as ‘three minutes’ ( Davenport, Honigman, & Druck, 2008). Especially true in that setting as well, the disorders which need to be reliable diagnosed or ruled out for each of these more frequent complaints in that setting can often be indicated by specific and pertinent elements of the history, when they are first learned then combined.

I suggested in that earlier short paper that teaching the chief complaint driven and focused history would be beneficial in the development of expertise in clinical reasoning. One of the largest challenges which the student must overcome to be able on day one to obtain and present a focused history, physical and differential is that “the ability to determine *pertinent* information (italics mine) is difficult for the student physician ( Davenport, *et al*., 2008, P 685). Those authors suggested that one way for the student to determine pertinence is to have a short differential diagnosis for the chief complaint. It has been shown that the most important determinant of whether the correct diagnosis is eventually reached is whether that diagnosis was on the list of those initially considered ( Mandin, Jones, Woloschuk, and Harasym, 1997).

The purpose of the current paper is to expand and develop the proposed method outlined in the earlier paper, to provide a more robust development of the findings in medical education literature which supported it, and to offer more specific and teachable examples for some presentations to the emergency department. The author believes that the focused method of teaching will generalize to other patient care venues as well.

## Development of Expertise: Review of Literature

How does a student learn to obtain the history and physical, consider a cogent differential diagnosis, and determine what is and is not pertinent so as to be able to present the case in the ED in 3 minutes? In other words, how does the novice, the student, become an expert in these, among the first of the now expected to be “entrustable” activities ( AAMC, 2014)?

Challenges have been identified (Norman, 2005) in finding unity and common language in the investigation of the concept of clinical reasoning, and even in agreeing what such reasoning is. Work in these areas has been published in such diverse fields as medical education, cognitive, clinical and social psychology, sociology and information processing. However, germane to the president effort, the majority of work has been focused on the ability to arrive at a diagnosis.

There exists a long and robust history of efforts to determine how expertise is developed in clinical reasoning. It was shown over six decades ago ( Rimoldi, 1961), in a simulation test of diagnostic skills, that experienced clinicians would ask more *useful* questions than junior students, demonstrating an effect of learning and experience to develop “a greater ability of expert clinicians to selectively attend to relevant information and narrow the set of diagnostic possibilities” ( Patel, Arocha, and Kaufman, 2001). In actual practice physicians have been shown to develop and test specific hypotheses early in the diagnostic interview ( Barrows, Norman, Neufeld, and Feightner, 1982) rather than to gather a complete comprehensive body of data. Particularly relevant to emergency medicine, Barrows, *et al*. (1982) found that experienced clinicians do not lose diagnostic accuracy even when the time to gather data is limited to ten minutes. It is a challenge to bring a novice student to the point where that may be true.

## Hypothetico-deductive reasoning and problem solving

One initial approach to problem solving ( Simon and Newell, 1971), of which clinical reasoning can be considered a type, was framed in the language of information processing. They proposed that “a few gross characteristics of the human information-processing system are invariant over task and problem solver” (Page 148). Elstein, Shulman, and Spafra (1978), pioneers in the use of cognitive science in the investigation of clinical competence ( Patel, *et al*., 2001), suggested that both experts and novice clinicians work through the early development of diagnostic hypotheses which they then use to account for clinical findings. This problem solving method was referred to as “hypothetico-deductive” reasoning ( Elstein, *et al.*, 1978; Patel and Groen, 1986). The clinical reasoning process was called “a problem solving process designed to adapt to the need to obtain more information to solve an *initially ambiguous diagnostic situation* and the need to work with a progressive unfolding of information over time” ( Barrows and Feltovich, 1987, p. 88, italics mine).

It was soon concluded that a general process of hypothetico-deductive reasoning would not get very far without being backed up by an accurate, and well organized medical content ( Barrows and Feltovich, 1987; Patel and Groen, 1986; Schmidt, Norman, and Boshuizen, 1990). Studies summarized in several reviews ( Berner, 1984; Mcquire, 1985; Mandin, *et al*., 1997; Coderre, Mandin, Harasym, and Fick, 2003; Norman, 2005) demonstrated that clinical reasoning was not the result of a *general process* of problem solving, but rather that it was specific knowledge domain dependent. It required specific cognitive processes for specific tasks ( McQuire, 1985), and was found to be knowledge based and experience specific ( McQuire, 1985; Schmidt, *et al.*, 1990). As Elstein and Schwartz later expressed it ( Elstein and Schwarz, 2002, p. 729) “It appears that diagnostic accuracy does not depend as much on strategy as on the mastery of content.”

Exactly which ‘domain specific knowledge’ was useful in clinical decision-making became an area of investigation. Medical education has traditionally begun, at least in the first two pre-clinical years, with an extensive preparation in the basic biomedical sciences, such as anatomy, biochemistry, pathology, pharmacology and physiology, all of which are required long before students start to decipher clinical problems. It was soon shown that as students progress in their education, the relevant knowledge base employed switches over from initial basic science to a more clinical knowledge base ( Patel and Groen, 1986; Patel, Evans and Groen, 1989; Schmidt *et al*., 1990). It has been noted that on the ward, in the clinical setting, “recall of basic science knowledge from the classroom is often slow, awkward or absent” ( Bowen, 2006, p2217).

Schmidt and colleagues (1990) proposed a step-wise development of clinical reasoning, in which the domain specific knowledge use by novices to approach clinical problems was rooted in the application of the basic sciences learned in the first pre-clinical years of medical school, namely rooted in anatomy, pathophysiology, biochemistry and so on. Experts, however, were found to make less use of basic biomedical science in daily reasoning ( Patel, *et al*., 1989 **;** Schmidt, *et al*. 1990; Boshuizen and Schmidt, 1992; Schmidt and Boshuizen, 1993). The second stage therefore of Schmidt and colleague’s (1990) proposed development of clinical reasoning invokes the development of models, representations of patients and their disease presentations, often referred to as “illness scripts” ( Barrows and Feltovich, 1987), of which we will say more soon.

## How experts structure and retrieve relevant information

Inquiry turned to examinations of how experts differ from novices in those elements of a presentation to which they attend, and remember (Norman 2005), and how they learn, structure and retrieve relevant information ( Mandin, *et al,*, 1997; Norman, 2005). Hobus, Schmidt, Boshuizen, and Patel (1987) compared case relevant recall between expert and novice clinicians, and found that experts had more accurate initial diagnoses, not surprising, but again particularly relevant to emergency medicine and most importantly, that while experts do not recall more *total* information about a presentation, they recall more *relevant* information. This echoes again those very early findings of Rimoldi (1961) demonstrating that the experts remember more useful bits of information, that is, clinical facts of greater pertinence. This leads us back to ask how experts develop the superior ability to discriminate relevant from less relevant data. In the words of Evans and Gadd (1989), “Experts are experts not only because they know more, but because they know differently. They have internalized strategies for managing and evaluating information..” ( Evans and Gadd, 1989, P. 211). Experts make better selective use of data, choosing relevant over relevant to retain, retrieve and apply ( Patel, *et al.*, 1989).

Schmidt and colleagues’ proposed that the most advanced stage of cognitive development in decision making (1990) invokes the use of ‘exemplars’. The expert has encountered a particular clinical condition enough times to have developed a large body of known examples, and can thus compare the present instance to the known pattern. In certain situations, this pattern of cognitive reasoning has sometimes also been referred to as pattern recognition, or as “non-analytical reasoning” ( Eva, 2004; Norman, Young, and Brooks, 2007). It likely occurs in those diagnostic activities which emphasize visual recognition, such as radiology ( Lesgold, *et al.*, 1988) dermatology, ( Norman, Coblentz, Brooks and Babcook, 1992), with EKG recognition including contextual features with visual recognition ( Hatala, Norman and Brooks, 1999; Norman, *et al.*, 2007; Ark, Brooks and Eva, 2006,). However, Barrows and Feltovich (1987) are of the opinion that it would trivialize a more complex process to claim that ‘pattern recognition’ is the main form of clinical reasoning for more complex clinical diagnostic situations. After all, they point out, most of the information needed to form a pattern is not available at the start of a clinical interview, so there remains the need to explain the process by which a clinician *seeks and organizes the information* from the clinical history to form a pattern.

It may be that for some more common or more straightforward presentations, an initial few bits of information (as in that old show “Name that Tune”!) serve to suggest a pattern, especially for a diagnostic entity frequently seen, such as biliary colic or renal colic. When we see that occurring, what has most likely occurred is that one or two elements of the presentation (e.g. right upper quadrant pain radiating to the back) combines with a relatively accessible contextual background (patient of proper age, gender and habitus) to both combined suggest a series of hypotheses and narrow them sufficiently that one or two more “notes” may be enough to “name the tune” with a high degree of certainty. We see this difference in reasoning styles when a clinician virtually immediately identifies, say, crushing chest pain and dyspnea in a hypertensive 60 year - old smoker, but takes a longer more systematic approach to a complex acid-base problem. Eva (2004) considers pattern recognition and hypothesis analysis both to be important aspects of the diagnostic decision, and current literature on the so-called “Dual Process theory of clinical decision making sees the forms of cognition also as complementary (see Palaccia, Tardif, Trilby, and Charlin, 2011). More to our point, it remains to explain how this process can be taught and acquired.

## Scripts, schema, semantic networks and cognitive structures

One must learn, at first explicitly, to apply certain reasoning patterns to organize the gathering of patient information and relating to it to an organized knowledge base ( Bordage and Lemieux, 1991; Custers, Regehr and Norman, 1996). It has been observed that “the difference between ‘good’ and ‘not so good’ diagnostic thinking cannot be expressed exclusively by the amount of knowledge one stores in memory but is expressed by the appropriate use of semantic” (meaning related) “formal strategies to learn and organize new concepts using abstractions and oppositions” ( Bordage and Lemieux, 1991, pg. S108)

A broad lexicon has been invoked to characterize what one might call a ‘cognitive architecture of clinical reasoning’, (a term I borrow from artificial intelligence based models ( Franklin and Strain, 2010, Qiao, *et al.*, 2014). This broad umbrella is broken up to include three general formulations.

“Semantic networks” ( Schmidt, *et al.*, 1990: Bordage and Lemieux, 1991: Arocha, Wang, and Patel, 2005), view knowledge and data as organized into units of meaning, sometimes called nodes ( Custers, Regehr, and Norman, 1996). These units of meaning are visualized as being connected together by links to form larger and larger units of meaning, sometimes called chunking ( Miller, 1956). Linking, combining and progressively organizing data and knowledge into larger units of meaning ties smaller units of meaning together, associatively, causally or hierarchically, to form higher units of meaning ( Evans and Gadd, 1989).

Another term, ‘Schema’ ( Brawer, Witzke, Fuchs, and Fulginiti, 1988; Coderre, *et al.*, 2003) has been defined, for example, as “a prototypical knowledge structure which enables patterns of relevant facts to be filtered out” ( Kushniruk, Patel, and Marley, 1998, p. 256). This postulates, essentially, a mechanism to explain the expert’s ability to attend to the most relevant data in which the expert has a more complete understanding of the larger constructs underlying a particular set of symptoms or signs.

One proposed benefit of organizing a large body of information using schema is that it might reduce the large burden of information or “cognitive load” ( Sweller, Ayres and Kalyuga, 2011) that can, in complex situations, overtax memory structures ( Qiao, YQ, *et al.,* 2014). The use of schema in instruction has been associated with improved retention of clinical knowledge ( Mandin, *et al.*, 1997; Blissett, Cavalcanti, and Sibbald, 2012). We will give an example of this schema utilizing approach later by way of example.

A “script” (Shrank and Abelson, 1975; Schrank and Abelson, 1977; Barrows and Feltovich, 1987; Custers, 2015), is described as a “(cognitive) structures that describes an appropriate sequence of events in a particular context”, this concept being extended to the description of a disease presentation or “illness script” ( Custers, Boshuizen, and Schmidt, 1998; Bowen, 2006; Lee, *et al.* 2010, Custers, E, 2015). A very comprehensive and inclusive review of the development of Script theory has been recently published, ( Lubarsky, Dory, Audetat *et al.*, 2015) which reviews the broad applications of scripts.

The invocation of these three separate but analogous notions of structure starts with the view that “there must be something in the way that knowledge is structured that enables the experienced clinician to solve medical problems quickly and accurately” (Custer, *et al*., 199s, P S55).

## Problem Representation

Whether invoking any of the above mentioned distinctions in title or nomenclature, the process of applying such knowledge structures to clinical data can be conceptualized as starting with what has been called the ‘problem’ representation ( Chang, Bordage, and Connell, 1998). Problem representation has been sometimes defined as “a short summary defining the specific (patient’s) case in abstract terms” ( Bowen 2006, p 2218). It is a conceptualization that was early applied to the teaching of mathematics ( Mayer, 1989), and in learning how to solve problems in physics ( Chi, Feltovich, and Glaser, 1981). Before that, the concept was invoked in early descriptions of reasoning in chess (DeGroot, 1965). Pioneering efforts to investigate clinical problem solving ( Simon and Newell, 1971) borrowed the chess metaphor of an initial approach to the problem solution with an internal representation or mental image. They characterized the problem solver as “representing the problem as a ‘problem space’ of possible solutions. In describing the role of the problem representation in clinical reasoning, and placing it within a framework of a cognitive structure of diagnostic decision making, Arocha, Wang and Patel (2005) stated that, “the formation of a good problem representation is critical in generating correct and effective decisions” (pg.157). They described the process as constructing “an ideal somewhat abstract model of the problem” (p 160). They characterized the development of the problem representation as one of organizing knowledge into meaningful structures in memory. Knowledge retrieval structures are thought to play a pivotal role in filtering irrelevant information and reinforcing the relevant associations (McGuire, 1985; Bordage and Lemieux, 1991; Custers, *et.al.*, 1996; Norman, 2005). Developing a cogent problem representation allows students becoming experts to better access and retrieve knowledge, and “problem representation is exemplified by the ability to transform clinical data into sets of *relevant”* structures ( Chang, *et al.*, 1998, p S110, italics mine).

## Semantic Qualifiers

In order to develop a working understanding of knowledge structure and to effectively use it in teaching clinical reasoning it is necessary to begin to understand some of the underlying elements on which such structures are built. Feltovich *et al*. (1984) spoke of ‘logical competitor sets’, that is, a set of plausible but easily confused diagnostic entities, conditions which might, on initial consideration account for a pattern of symptoms and signs. Kushniruk *et al.,* (1998) has called this a concept of “small worlds”, and referred to experts as organizing the retrieval of relevant information of disease categories forming subsets (“small worlds”) of “logically related and distinguishable diagnostic hypotheses..allowing them to focus on the few critical findings that clearly differentiate between competing hypotheses” (p.257).

How do we choose, determine and define those few critical findings? Bordage and Lemieux (1991) introduced the idea of “semantic qualifiers”. Semantic qualifiers represent an abstraction of clinically relevant elements of the history (and later physical exam and labs), into relevant symptom variables to be framed as “oppositional relationships”. These symptoms or aspects of the history can thereby be framed as binary, paired, or opposing elements of a complaint. They are dichotomies that can be used to compare, contrast, and draw distinctions among diagnostic possibilities ( Bowen, 2006). Using this structure thereby characterizes and describes any clinical complaint along a number of different dichotomous axes (Chang, *et al.,* 1998, Bowen, 2006). For example, a pain might be of *either* acute *or* of chronic duration, it may be *either* continuous, *or* intermittent,localized *or* diffuse, severe or mild, and so on. One example has made of an acutely painful swollen knee as being acute and mono-articular swelling of a large joint ( Bordage and Lemieux, 1991; Bowen, 2006).

Semantic qualifiers, dichotomous descriptive axes can be used to characterize the nature of most, if not all chief complaints. Consider, for example, the chief complaint of “chest pain”, which is one of the most frequently encountered reasons for a visit to the emergency department. The chest pain can be either acute or chronic, of new onset or recurrent, sharp or dull, radiating to sites such as neck, jaw or arm pertinent to coronary artery disease or not. It can be associated with dyspnea or not, and occur in the context of multiple risk factors or not. These axes, dichotomous, or in some cases only relatively dichotomous (e.g. how *many* risk factors) are of key importance, even to represent the problem adequately, and certainly in the formation of clinical meaning in the form of semantic networks and illness scripts.

The importance of utilizing specific distinctions, specific dichotomous distinctions between elements of the presentation of similar diseases, is the key cognitive skill required to learn to recognize and diagnosis clinical presentation. Nendaz and Bordage (2002) were able to demonstrate that it is possible to teach medical students to develop conceptualization using semantic qualifiers, however this process alone was not sufficient to improve diagnostic accuracy. In developing the clinical presentation curriculum at the University of Calgary, Mandin, *et al.*, (1995) found that utilizing a ‘compare and contrast’ modus of instructing students in recognizing disease presentations was superior to independently learning to recognize diagnostic entities. Focusing on the *dichotomies* between presentations, allowing and teaching students to discriminate between diseases, using the dichotomous semantic qualifiers detailed above, is how they learn to separate disease presentations. It is this very process which the presently described chief complaint differential diagnosis guided HPI is meant to utilize.

## The chief complaint driven history, semantic qualifiers and expertise

Figure 1 in an earlier section expressed a conceptual distinction between traditionally taught method of obtaining a medical history and the present proposal. Students are traditionally taught is to acquire elements of the history *in sequential separate categories* ( Bickley, Szilagyi, and Bates, 2007; DeGowin and Brown, 2007; Swartz, 2002).

I have here reviewed evidence that it is from learning to gather and separate *relevant or pertinent* information which has for so long been to separate the expert from the novice.

In using a chief complaint driven history, each potential diagnosis in the differential generates in the mind of the increasingly experienced clinician a series of questions than can help either move confirm, rule out, or at least make more or less likely, that diagnosis. These questions can be characterized, for the most part, as described above as dichotomous semantic qualifiers ( Bordage and Lemieux, 1991; Bowen, 2006). By using these dichotomous axes to separate among the possibilities, moving toward or away from a diagnosis, the expert clinician ultimately creates an increasingly likely differential, and allows for better prioritization of steps in the further diagnosis and treatment. By asking students specifically to use specific questions pointing toward or away from a diagnostic possibility, we are asking them to find specific differentiating “semantic” qualifiers to compare and contrast potential diagnoses for a given complaint.

This process, having become almost second nature to an experienced clinician can be explicitly taught to a junior clinician or a student. The student can be taught to take each entity in the differential diagnosis, and think even from the beginning of the characterization of the chief complaint to ask a few questions which would help move one’s thoughts towards or away from each diagnostic entity. These specific questions are derived from relevant semantic qualifiers, dichotomous axes, specific differentiating elements of the history, past or present, which have been found to allow one to separate or discriminate among competing diagnoses. In short, the experienced clinician would, rather than conduct a comprehensive review of the past medical history, instead seek for and select a series of specific pieces of information to inquire about immediately relevant elements. These would come from, *and lead with experience to the development of*more sophisticated “illness scripts”.

## The Example of Abdominal Pain

The causes of abdominal pain differ depending on where exactly in the abdomen pain is located, so the first question to ask any patient who presents with abdominal pain is “Where exactly in the abdomen is this pain located?” In the case of mid-epigastric pain, for example, there are a number of clearly separate causes for such pain ( Silen, 2010). Developing a differential diagnosis of the most likely causes for this particular patient’s mid-epigastric pain is useful in determining priorities for laboratory and imaging studies.

Table 1 demonstrates the use of the chief complaint driven history using the example of mid-epigastric pain, and illustrates the use of specific dichotomous axes, “semantic qualifiers”, if you will, to ask specifically targeted questions meant to separate, quickly and directly, among the more likely diagnostic entities. The answers to these specific questions will help the clinician prioritize more or less likely causes for the patient’s pain. Additionally, using specific questions will help the learning clinician to develop a knowledge and experience of those aspects of the history which help define a particular disorder, and help the student develop an “illness script” to help recognize the disorder when seen the next time.

Inspection of the table shows a number of possible diagnostic entities to account for possible pain. In keeping with the basic tenants of emergency medicine, where a “rule out worse case scenario is almost pathognomonic of decision making in the ED ( Croskerry, 2002, p 1186) the more dangerous possibilities are considered first (pancreatitis, biliary tract disease and obstruction) before the more common GERD.

**Table 1.** Using the Chief Complaint Driven History to Diagnose Abdominal Pain

Initial *Characterization*: Site, onset, Character, Radiation, Associated features, Timing, Exacerbating factors (“SOCRATES” or other mnemonic to better describe or characterize the chief complaint)		
Differential Diagnosis/Possible Cause	Differentiating feature (Binary Semantic Qualifier)	Question to ask in taking history
***MIDEPIGASTRIC PAIN***		
Pancreatitis	Radiation to back (Yes or no)	Does the pain go to the back?
	Prior history of pancreatitis (Yes or no)	Have you ever been told you have pancreatitis
	Major risk factors, alcohol, biliary tract, ( Yes or no)	Do you drink alcohol. How much would you say you drink?
		Have you every had gallstones or gallbladder disease?
	Are there other specific risk factors ( Yes or no, and what)	(May be other specific factors such as drugs, hypercalcemia, scorpion stings, etc.
Biliary Tract Disease, Gallstones, biliary colic, acute cholecystitis and others	History of gallstones (Yes or no)	Have you every had gallstones or gallbladder disease?
	Jaundice (Yes or no)	Have you ever had dark urine, or yellow eyes? How about light stools?
Epigastric presentation of appendicitis (early phase)	Migration of pain (Yes or no)	Does the pain stay right there or is it moving somewhere in the abdomen?
	Change in appetite (Yes or no)	How has your appetite been?
	History of appendicitis (Yes or no)	Do you still have your appendix?
Early presentation of obstruction	History of abdominal surgery (Yes or no)	Have you every had any abdominal surgeries?
	Biliary vomiting (Yes or no)	Have you been vomiting? What color is it?)
	Peritoneal signs (Yes or no)	Does it hurt you to walk? Did it hurt you to ride here in the car?
GERD, Gastritis, Ulcer, and related	History of GERD (Yes or no)	Have you ever had an ulcer, reflux or gastritis?
	Relationship with food (Yes or no)	Have you ever had an endoscopy?
		Does food make the pain better or worse?
Non-abdominal causes of epigastric pain	Cardiac symptoms (Yes or no)	Is this pain in your chest? Do you feel short of breath? Do you have any cardiac history? How about (risk factors) HTN, DM, cholesterol? Do you smoke? Do you have a family history of heart attacks?

We can see from inspection of the table that in considering several diagnostic possibilities, the clinician may choose from a few high yield questions to help make one diagnosis more likely than another. These questions are based, as can be seen, along the lines of dichotomous and differentiating units of meaning, the so-called “sematic qualifiers”. The intention of teaching this method of reasoning is that the novice clinician will soon acquire an arsenal of specific questions helping to differentiate among competing diagnoses and move more quickly and effectively toward the more likely diagnoses.

## The case of altered mental status, using a “schema” approach

Mandin, *et al.,*(1997), Coderre, *et al*. (2003), and Blissett, *et al*. (2012) have been among those demonstrating that in areas of medical knowledge with multiple and complex factors connected with a clinical diagnosis, having a cognitive map or schema allows for better problem solving, learning and retention. Schemas are thought to improve knowledge organization, and would thus be expected to be helpful in situations in which multiple dimensions may come into play. Mandin, *et al*. (1997) illustrate the concept of schema based education utilizing the model of worsening renal function (p.178). In Blissett *et al*.’ *s* (2012) study, the complexities of cardiac auscultation and diagnosis was better taught using a schema approach than a more traditional method.

**Table 2.** Using a Schema Approach for the History of Altered Mental Status

Clarifying what “Altered Mental Status means”	Differentiating feature (Dichotomous)	Question to ask in taking history
**Coma**		Very important with ***any*** of these varieties of “altered mental status” to establish what the patient’s baseline is. Are they normal, do they work, do they speak, make sense, feed themselves, interact, take care of their own finances, watch TV, or need care with ADLs? Establish baseline then variation from it.
**Lethargy**		
**DELIRIUM, Dementia, psychosis**		
**Using Schema of “classes of causes” to evaluate possible diagnosis**		This is using a schema or organizing approach along the lines of what “classes of disorders” can have altered mental status as their final common result.
**Structural causes**		
Stroke	Focal Deficit	Do you (Does the patient) have any difficulty with speech or vision? Difficulty moving or talking. Walking?
Bleed	History of Stroke or TIA	Have you ever had a stroke or a TIA?
	Sudden Onset	Did these symptoms come on suddenly or more gradually?
	Headache, vomiting	Have you had a headache?
	History of anticoagulation	Are you (the patient) taking any blood thinners?
**Infections**		
CNS infection, meningitis, encephalitis	Fever or chills	Have you had any fever or chills?
	Stiff neck	Any headache. Have you had a stiff neck?
General sepsis, pneumonia, UTI, cellulitis, other forms of infection	Focal factors	Have you had any burning on urination? Cough, chest pain, shortness of breath. Have you had pain on urination or a strange smell to your urine? Any areas of inflammation in the skin?
**Metabolic causes**	Diabetes, hyper- or hypo-glycemia	Do you have diabetes? Have you ever had low blood sugar? Ever been told you have problems with your kidney function? Have you ever had problems with your thyroid?
	Electrolyte abnormalities, hypercalcemia, etc.	
	Renal failure	
	Thyroid Storm	
**Ingestions**	Intentional or unintentional	Have you taken any medications or drugs which were not prescribed? Do you use alcohol or drugs? Are you on any ***new medications?***
	Drug interactions	Have you taken new medications or other medications than usual?
**Seizure Activity - Post ictal**		Does the patient have a history of seizures? Any observed seizure activity?
**Progressive neurologic diseases**	Family history, speed of onset, other related features	Does the patient have a family history of similar disorders? Has the behavior or function changed gradually?
**PSYCHIATRIC CAUSES (after you have considered physical causes)**	Psychiatric historyRecent social stressors	Does the patient have a psychiatric history?Are there recent changes in the patient’s life which could lead to increased emotional stress?

Inspection of the Table 2 shows that first, the phrase “altered mental status” must be characterized to determine whether, broadly speaking, this means frank coma or not. Frank coma falls within another diagnostic category than the change in behavior, alertness and responsiveness for which medical student or novice learner would generally be applying diagnostic reasoning, and so will not be considered at the primary focus here.

For those patients with the less acute variation of altered mental status, stupor, decreased responsiveness, change in behavior, “just not right”, and those commonly encountered presentations, it would still be very challenging to remember and apply a simple list of differentials in the same manner as was illustrated in the example of abdominal pain, in which there are a relatively finite number of diagnostic entities which present with mid-epigastic pain.

For altered mental status, using a schematic approach, in other words applying an organizational scheme to the classes of causes, a final common pathway of which can be a subtle but definite change in sensorium, allows the learning to clinician to consider diagnostic pathways. Is this a structural abnormality of the brain, a bleed or a stroke? Can we derive a few questions which might point in that direction? Or is this a manifestation of infection, or sepsis? What are a few questions which might lead in that direction? Using these schematic organizational guidelines for altered mental status modifies our chief complaint driven differential diagnostic guide approach to the history, but the essence of the focused diagnostic inquiry does not change.

## Conclusion

A focused approach to teaching the medical history, previously proposed in a short outline has been further developed here. After reviewing a selection of what has been learned regarding the cognitive science of medical education, the chief complaint driven approach is proposed as a teaching device as well as a clinical tool. Framed within the context of a decades long inquiry and research into medical information processing and decision making, it is proposed that this teaching method combines what has been learned in cognitive research to lead to a focused, direct and effective medical history within the time demanding context of the emergency department. It is conceivable that this direct and focused approach, might prove useful in other clinical contexts as well.

## Take Home Messages

Teaching students to approach a patient history through a chief complaint differential diagnosis and focused history develops clinical reasoning. This approach is consistent with a long and robust research into the development of clinical expertise as demonstrated in several lines of inquiry into cognitive processes. The history of research is reviewed and shown to contribute directly to the development of a chief complaint driven history. This approach is useful both as a teaching device and a clinical approach to patients in the emergency department as well as other venues of care.

## Notes On Contributors

Dr Nierenberg is a member of the core faculty in emergency medicine at the Hackensack University Hospital and an assistant professor at Hackensack Meridian School of Medicine at Seton Hall University. He previously spent five years as Hackensack’s clerkship director in emergency medicine for St. George School of Medicine. He has training and board certification in emergency medicine, internal medicine, and critical care. His ORCID number is https://orcid.org/0000-0001-7014-2598.
