# Supplementary material for: Using the Chief Complaint Driven Medical History: Theoretical Background and Practical Steps for Student Clinicians
Source: MedEdPublish (2016). 2020 Jan 20;9:17. [Version 1] doi: 10.15694/mep.2020.000017.1 (PMC10734793; doi:10.15694/mep.2020.000017.1)
Supplement: Peer review history

# Reviewer report: Reviewer response for version 1

**DOI:** 10.21956/mep.19980.r30388

**Published:** 2020-11-16

**Authors:** Barbara Jennings

**Affiliations:** 1 Norwich Medical School

This review has been migrated. The reviewer awarded 4 stars out of 5 This is a thoroughly researched article that cites a large body of primary and secondary literature about the differences between how novices and experts learn, retain, and apply information (e.g. from biomedical sciences) to clinical reasoning. The author presents an approach for teaching a focused medical history in an emergency room, and the description of explicit examples follows a detailed discussion of what the author refers to as “the cognitive architecture of clinical reasoning.” I am not a clinician, but read this from my perspective as a problem-based-learning tutor and scenario-writer. My intention is always to keep the learner’s eye on the presenting complaint and to support hypothesis generation about the differential diagnosis. This article made me think about ideas of “dual-processing” with pattern recognition working in parallel with hypothesis analysis.I found the article very interesting and there is plenty of scholarship available via the extensive bibliography which many readers might use. In places is it is not an easy read, which is not surprising given its scope and the fact that many decades of research about cognitive science and medical education is described. I think the transitions between background information and the method of instruction proposed may be a bit abrupt. Also, some abbreviations and terminology linked to figure 1 could have been explicitly described in a legend, to make the section and theories easier to navigate.I do think many clinical educators and educationalists will find this article useful. In particular, those responsible for clinical teaching; script concordance testing; evidence based medicine; and curriculum design that includes the virtual or simulated patient.

Reviewer Expertise:

NA

No decision status is available

# Reviewer report: Reviewer response for version 1

**DOI:** 10.21956/mep.19980.r30389

**Published:** 2020-01-21

**Authors:** Balakrishnan Nair

**Affiliations:** 1 Centre for Medical Professional Development and University of Newcastle

This review has been migrated. The reviewer awarded 5 stars out of 5 I enjoyed reading this well researched article. It provides the context and evidence behind the history taking for arriving at a timely diagnosis. Experts arrive at the diagnosis with a hypothetical-deductive process combined with illness scripts. This is like starting to examine the lower limbs when the patient presents with lower limb weakness and back pain , whereas the novice may start with cranial nerves !It is always good to have a working diagnosis with at least few differential diagnosis to minimise diagnostic errors .

Reviewer Expertise:

NA

No decision status is available
